# Mortality of Infectious Diseases

**Published:** 1903-12-05

**Authors:** 


### The Hospital.

A JOURNAL OF

The Medical Sciences and Hospital Administration.

VOL. XXXV.—No. 897. DECEMBER 5, 1903.

### MORTALITY OF INFECTIOUS DISEASES.

THAT inexhaustible storehouse of knowledge, the annual Report of the Local Government Board, from which we derived last week an account of the war perpetually being waged between analysts and adulterators, affords equally important and interesting information concerning the actual mortality from infectious diseases, information which by and bye will serve effectually to display the value of any improvements in treating them that from time to time may be introduced. The figures available for this purpose are now sufficiently large, and the area from which they are derived is now sufficiently extensive, to exclude the errors which are always liable to follow from the e[illegible]ployment of a limited number of instances for statistical purposes. Alone among urban registration districts, Aldershot, with a population of 32,239; Maidstone, with 28,457; Cheltenham, with 50,047; and Blackpool, with 53,727, have not authorised the publication of the figures. This trivial recalcitrancy has not prevented the Board from obtaining and circulating the weekly returns from the remaining 245 urban sanitary authorities, representing an aggregate population of more than 19,000,000; so that the absence of returns from four only, with an aggregate population of 164,470, is not likely in any way to vitiate the general conclusions. The figures are, of course, for the year 1902; and they show 13,976 cases of small-pox, of which more than half, or 7,796, were in London, 228 in Erith, 701 in Edmonton, 481 in Tottenham, 211 in East Ham, 86 in Leyton, 146 in Walthamstow, 815 in West Ham, 560 in Liverpool, 178 in Oldham, and the rest scattered over the country in comparatively small numbers at any place. The total mortality was 2,062, or 14·8 per cent. of the occurring cases. These combined figures of prevalence and of mortality, in the case of an absolutely preventable disease, cannot be described as other than appalling, and they furnish additional and imperative reasons for that early reconsideration of the vaccination laws which would in any case be rendered necessary by their temporary character, and by the supineness of a large number of local authorities in enforcing them, or, to speak more correctly, in failing to enforce them. It has been conclusively shown, in the last report of the Medical Officer of the Local Government Board, as distinguished from the report of the Board itself now under discussion, that the "conscientious objector" scarcely counts at all in the matter, and that the existence amongst us of an unvaccinated population, as a perpetual menace to good citizens, is almost entirely due to official neglect, as distinguished from individual opposition.

Diphtheria, including membranous croup, was more than twice as prevalent as small-pox, and precisely equal to it in its percentage of mortality. 34,268 cases were notified, among which there were 5,063 deaths, but the disease was far more generally diffused than small-pox. London was answerable for over ten thousand cases, Brighton for 435, Portsmouth for 495, Bristol for 1,095, Birmingham for 720, Liverpool for 1,048, Leeds for 639, Sheffield for 969, and Cardiff for 686—the comparatively few cases notified as membranous croup being omitted from this enumeration. It would be very interesting, if the facts were available, to compare the mortality of districts in which antitoxin was early and freely used with that of others; but of this there is no present possibility. Among low mortalities we notice Brighton, with 36 deaths in 437 cases, or little more than half the average; Eastbourne, with three deaths in 52 cases; Broadstairs, with one death in 25 cases, and so on. The rate of mortality at Portsmouth was nearly double that at Brighton, and at Reading 23 cases died out of 60, as against seven out of 58 at Edmonton. Such variations may no doubt be due to different grades of severity in the disease, or to different degrees of strictness in the enforcement of notification; but none the less they require to be explained. It does not seem reasonable that the chances of recovery from a common malady should be so very much greater in one locality than in another. Of all the notifiable diseases, scarlet fever was the most frequent, and was accountable for nearly a hundred thousand cases, but the resulting mortality was only 3·7 per cent., or absolutely only one-third greater than that from small-pox, and about three-fourths of that from diphtheria. The fevers, chiefly enteric, but with a few examples of typhus, continued, and relapsing, were notified in over seventeen thousand cases, and were attended by a mortality of 55 per cent. for typhus, and a little under 16 per cent. for the other varieties. Puerperal fever was notified in 1,417 cases, and was answerable for 710 deaths. Nearly one-fourth of the cases (311) were in London, and were attended by the high mortality of 66 per cent. Other large numbers were 18 at West Ham, 13 at Devonport, 39 at Bristol, 35 at Birmingham, 15 at Leicester, 13 at Derby, 13 at Birkenhead, 70 at Liverpool, 47 at Manchester, 31 at Kingston-upon-Hull, and 37 at Sheffield. But the highest figures are furnished by Wales, and they are such as to call loudly for explanation. The Principality notified no fewer than 116 cases and registered 56 deaths. In other words, one twenty-sixth of the total population of England and Wales has had more than one fourteenth of the cases, and nearly one thirteenth of the mortality. Out of 18 Welsh urban districts only 6 have escaped; and at Rhonda, with a population of 113,735, there were 43 cases and 23 deaths. It seems certain that the practice of midwifery in Wales must be such as to call for the prompt action of the local authorities concerned.

